# Data quality within the Netherlands Heart Registration: Ready for prime time?

**DOI:** 10.1007/s12471-023-01802-2

**Published:** 2023-08-04

**Authors:** Niels M. R. van der Sangen, José P. S. Henriques

**Affiliations:** Department of Cardiology, Amsterdam UMC, University of Amsterdam, Amsterdam Cardiovascular Sciences, Amsterdam, The Netherlands

Public reporting of demographic, procedural and outcome data in patients with cardiovascular disease increases transparency and can enable an improvement in quality of care. The Netherlands Heart Registration (NHR) collects data regarding all invasive cardiac interventional, electrophysiological and surgical procedures in 71 hospitals, including 30 cardiac intervention and heart centres across the Netherlands. This information is also an important source of data for clinical research. Using real-world data has several advantages compared to using data from randomised controlled trials (RCTs). Foremost, RCTs usually have numerous inclusion and exclusion criteria and are therefore at higher risk of selection bias compared to registry-based studies. When using registry data, either for quality monitoring or clinical research, data completeness and accuracy are of the utmost importance.

In this issue of the *Netherlands Heart Journal*, Houterman et al. present the method and results of the data quality control system within the NHR using data from the NHR percutaneous coronary intervention (PCI) registry as an example [1]. Data validation and verification were performed using standard quality controls and monitoring visits. In 2019, a random sample of 50–100 medical records of patients treated with PCI in 2016 or 2017 was reviewed in each hospital by an independent auditor. In the NHR PCI registry, data completeness was high, being on average 99.4% complete for demographic data, 99.5% for procedural data and 99.4% for outcome data. Houterman et al. also reported that for all variables combined, the accuracy was high at 97.6% and almost all variables had an accuracy above 95%. In the Swedish nationwide SWEDEHEART registry, a comparable monitoring method is used by which several hospitals are randomly selected for monitoring visits each year. During these visits data entered into the SWEDEHEART database are compared to the medical records of 30–40 randomly selected patients per hospital. In 2007, 96.1% agreement was found in SWEDEHEART, comparable to the accuracy in the NHR PCI registry [2].

Still, accuracy for some variables in the NHR PCI registry dropped below the 95% threshold. Interestingly, multivessel disease was inaccurately scored in 9.3% of the 2622 records that were reviewed. Importantly, the variable multivessel disease has previously been used extensively in research conducted using NHR PCI registry data, both as inclusion criteria or as a (potential) confounder [3, 4]. The NHR should be commended for its efforts to improve the accuracy of the multivessel disease variable, especially since consistency between hospitals has already improved since clarification of the definition and the inclusion of various examples in the data dictionary. However, future monitoring will have to confirm if the accuracy of this variable can rise above the 95% accuracy threshold.

Although randomisation remains the only reliable way to control for confounding when comparing two (or more) groups, RCTs are associated with high costs, vast regulatory requirements and the previously mentioned risk of selection bias. Some of these limitations can be overcome by incorporating RCTs within registries in so-called registry-based RCTs. By including a randomisation module in registries with unselected consecutive enrolment, such as the NHR PCI registry, the advantages of an RCT can be combined with the strengths of an all-comers clinical registry. Hopefully, registry-based RCTs can increase trial efficiency and reduce costs. The NHR is already working on the necessary (governance and digital) infrastructure for registry-based RCTs by collaborating with other stakeholders in the Heart4Data consortium. At present there are still several hurdles to be taken before data from, for example, the NHR PCI registry can be used in registry-based RCTs. First, the baseline and procedural characteristics that have been collected so far are limited and although the most recent data dictionary of the NHR PCI registry (version 23.1.2) includes additional characteristics for chronic total occlusion PCI and PCI in cardiogenic shock patients, reporting of these data is non-compulsory. Second, all four clinical outcome measures collected in the NHR PCI registry [i.e. coronary bypass grafting within 24 h, myocardial infarction (MI) within 30 days, target vessel revascularisation (TVR) within 1 year and mortality] were highly accurate, but non-compulsory outcomes such as MI within 30 days and TVR within 1 year were complete in only 83.4 and 87.5% of patients, respectively. Also, other relevant outcomes are missing, such as MI beyond 30 days, stroke and bleeding. Other registries, such as the FORCE-ACS and ZON-HR registry, collect these outcome data, but require informed consent or use an opt-out procedure, which are inherently associated with some degree of selection bias [5]. Future linking of the NHR PCI registry with other sources of outcome data (e.g. data from the Dutch Hospital Data foundation) could therefore be beneficial, especially if combined with data regarding drug prescriptions.

In conclusion, the quality checks and monitoring process of the NHR PCI registry demonstrate that data completeness and accuracy are high. Still, some variables require specific attention, especially given that use of data from the NHR PCI registry and other NHR quality registries will likely increase in the future.
